# Adams–Oliver Syndrome Type 3: A Case Report of Concurrent RBPJ, CACNA1A, and Double-Heterozygous MTHFR Variants

**DOI:** 10.3390/diagnostics16020274

**Published:** 2026-01-15

**Authors:** Grațian Cosmin Damian, Valerica Belengeanu, Cristina Popescu, Diana Marian, Ramona Amina Popovici, Carolina Cojocariu

**Affiliations:** 1 Department of Medicine, Faculty of Medicine, “Vasile Goldiș” Western University of Arad, 94-96 Revolutiei Blvd., 310025 Arad, Romania; damian.gratian@uvvg.ro (G.C.D.); belvtim@yahoo.com (V.B.); 2 Faculty of Pharmacy, “Vasile Goldiș” Western University of Arad, 94-96 Revolutiei Blvd., 310025 Arad, Romania; pursega36@gmail.com; 3 Department of Dentistry, Faculty of Dentistry, “Vasile Goldiș” Western University of Arad, 94-96 Revolutiei Blvd., 310025 Arad, Romania; cojocariu.carolina@uvvg.ro; 4 Department I, Faculty of Dental Medicine, Victor Babes University of Medicine and Pharmacy of Timisoara, 300041 Timisoara, Romania

**Keywords:** Adams-Oliver syndrome (RBPJ), transverse terminal limb defects, CACNA1A, MTHFR, homocysteine, neuropsychiatric manifestations

## Abstract

**Background and Clinical Significance:** Adams–Oliver syndrome type 3 (AOS3) is a rare congenital disorder typically characterised by terminal transverse limb defects and variable involvement of other organ systems. Although pathogenic variants in RBPJ are well established in AOS3, associated neurodevelopmental or psychiatric features have been only sporadically documented. **Case Presentation**: We describe a male patient first evaluated at the age of 10 years and subsequently re-evaluated at 14 years, with AOS3 presenting terminal limb defects together with autistic-like behaviour, cognitive difficulties, dyslexia, and recurrent depressive symptoms. Whole-exome sequencing (WES) identified a heterozygous pathogenic variant in RBPJ (c.505A>G; p.Lys169Glu), confirming the molecular diagnosis of autosomal dominant AOS3. Additional findings included a heterozygous missense variant in CACNA1A (p.Arg1678Cys), a gene linked to neurological disorders with broad phenotypic variability. Because of elevated homocysteine levels, the patient was also tested for MTHFR variants and was found to be heterozygous for C677T and A1298C. **Conclusions:** This case illustrates a rare combination of a validated AOS3-associated RBPJ variant, along with additional CACNA1A and MTHFR variants that may influence the patient’s neurocognitive and psychiatric characteristics. The results underscore the importance of comprehensive genetic testing in atypical AOS presentations and highlight the complexity of interpreting overlapping genetic factors.

## 1. Introduction

Adams–Oliver syndrome (AOS) (MIM 100300) was first described in 1945 by Adams and Oliver in eight members of the same family who presented with aplasia cutis congenita (ACC) of the scalp and transverse limb defects [1]. AOS is an uncommon congenital malformation syndrome, with an estimated prevalence of 0.44 per 100,000 live births and approximately 1 in 225,000 individuals in the general population [2]. The condition is clinically heterogeneous, but it is characterised by scalp ACC, present in 75 to 85% of cases, transverse terminal limb defects (TTLDs), with prevalence of 85%, and cutis marmorata telangiectatica congenita (CMTC), described in 20–25% cases [3]. There may be other symptoms, but they usually affect the cardiovascular system (about 20% of the time) and less often the central nervous system, eyes, kidneys, and cardiopulmonary and gastrointestinal systems [2]. The clinical presentation varies from mild, localised limb anomalies to severe, occasionally life-threatening multisystem involvement [4]. Diagnosis is based on established clinical criteria that categorise findings into major (TTLD, ACC, and positive family history) and minor categories (CMTC, congenital heart defects, and vascular anomalies) [4,5]. AOS is predominantly transmitted in an autosomal dominant (AD) manner, although autosomal recessive (AR) inheritance and isolated instances have also been documented [2].

Genetic studies show that 36–48% of patients carry pathogenic variants in one of the known AOS-associated genes. The AD-associated genes include ARHGAP31, DLL4, NOTCH1, and RBPJ, while DOCK6 and EOGT are implicated in AR forms of the condition [6]. Among these, RBPJ is the defining gene for AOS type 3 (AOS3), and encodes the central transcriptional regulator of the Notch signalling pathway. Variants in RBPJ disrupt Notch-mediated transcriptional activity and have been linked to the characteristic pattern of ectodermal and limb involvement seen in AOS3 [2].

In parallel, pathogenic variants in the CACNA1A gene, which encodes the α1A subunit of the P/Q-type voltage-gated calcium channel, are associated with a broad spectrum of neurological and developmental disorders. These include episodic ataxia type 2 (EA2; OMIM 108500), characterised by recurrent episodes of ataxia, nystagmus and vertigo, and familial hemiplegic migraine type 1 (FHM1; OMIM 141500). Variants in CACNA1A have also been described in patients with developmental delay or intellectual disability, epilepsy, developmental and epileptic encephalopathy (DEE42; OMIM 617106), paroxysmal dystonia, and a range of neuropsychiatric manifestations. Considerable phenotypic heterogeneity and incomplete penetrance have been noted in affected individuals [7].

Another gene of interest in neurodevelopment and neuropsychiatric vulnerability is MTHFR (methylenetetrahydrofolate reductase). The MTHFR enzyme is essential in folate metabolism and in the conversion of homocysteine to methionine. Common functional variants, such as C677T and A1298C, reduce enzymatic activity and can lead to elevated homocysteine levels. Folate-dependent methylation plays a key role in the synthesis of neurotransmitters—including serotonin, dopamine, epinephrine and norepinephrine—linking MTHFR dysfunction to mood regulation, cognitive performance, and stress response mechanisms [8,9]. Although evidence remains inconsistent, MTHFR polymorphisms have been discussed in the literature as potential modifiers of folate metabolism that may be associated with certain neuropsychiatric or neurobiological characteristics [10].

In this context, we report a patient with clinical features consistent with AOS3 who carries a pathogenic RBPJ variant, together with a CACNA1A variant and heterozygous MTHFR variants. The coexistence of these findings may represent additional findings that could modify aspects of the phenotype (limb anomalies, cognitive deficits, and psychiatric symptoms) observed in this case.

## 2. Materials and Methods

### 2.1. Case Description

The patient is a male individual who was initially evaluated at the age of 10 years for shortening of the fingers and toes and subsequently re-evaluated at the age of 14 years. He is the first child of healthy, non-consanguineous parents, with an unremarkable family history. The pregnancy and delivery were uncomplicated. He was born at term with a birth weight of 3100 g, an occipito-frontal circumference (OFC) of 34 cm, and a length of 47 cm. Apgar score was 9. At birth, shortening of the fourth and fifth digits of both hands and both feet was noted and initially attributed to possible amniotic band syndrome.

**Figure 1:**
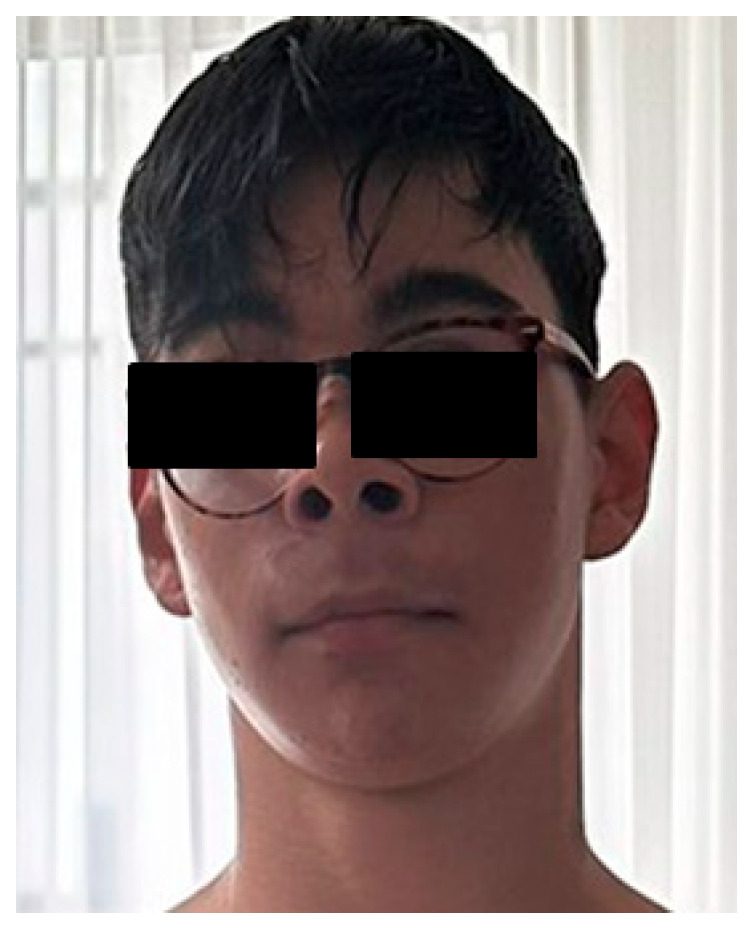
Facial appearance with subtle dysmorphic facial features.

During the first year of life, he experienced an episode of afebrile seizures. His medical history throughout childhood was notable for behavioural dysregulation, emotional instability, anxiety, attention deficit, hyperactivity, and inattention, for which he received psychotherapy. He also exhibited difficulties in social interaction and poor academic performance, with mild intellectual functioning (IQ 69), particularly affecting mathematical skills and was accompanied by dyslexia. No congenital heart disease was identified on cardiac ultrasound. Magnetic Resonance Imaging (MRI) of the brain did not reveal abnormalities. Aplasia cutis congenita (ACC), cutis marmorata telangiectatica congenita (CMTC), and other vascular anomalies were assessed by clinical and dermatologic examination and were not observed. EEG was normal and excluded epilepsy. At re-evaluation at age 14, the patient had appropriate growth parameters for age (height 165 cm; weight 50 kg) and a cephalic perimeter of 56 cm. Facial examination revealed no major dysmorphic features. He wore glasses for myopia and astigmatism and had a mildly depressed nasal bridge and low-set ears (Figure 1).

**Figure 2:**
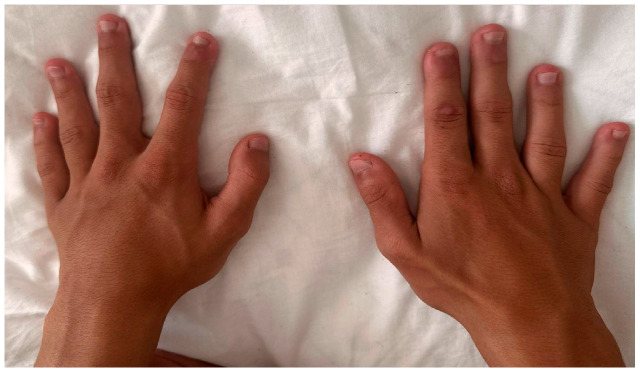
Hands with short fingers IV and V.

**Figure 3:**
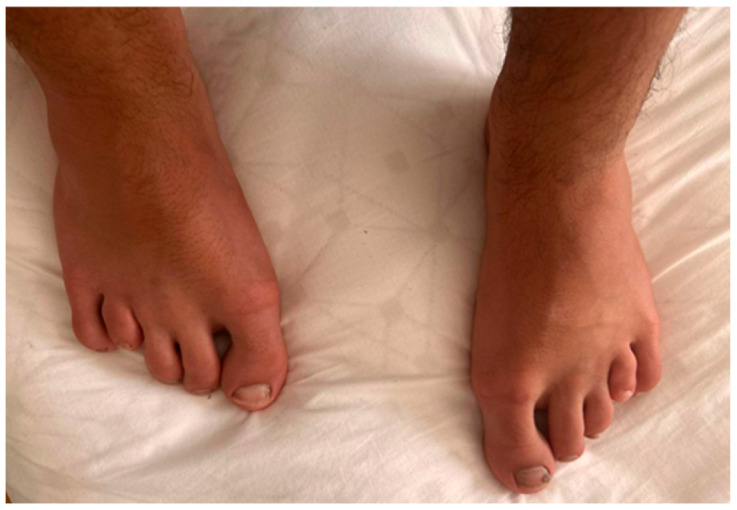
Bilateral hypoplasia in IV and V toes and small nails.

On physical examination, the fourth and fifth fingers of both hands were shortened (Figure 2), and hypoplasia of the fourth and fifth toes was noted bilaterally in the lower limbs (Figure 3).

**Figure 4:**
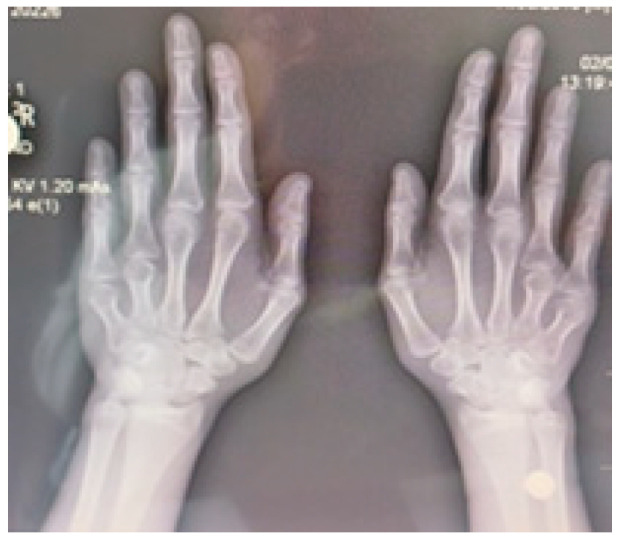
Radiograph of both hands demonstrating bilateral hypoplasia of the fourth and fifth metacarpal bones.

**Figure 5:**
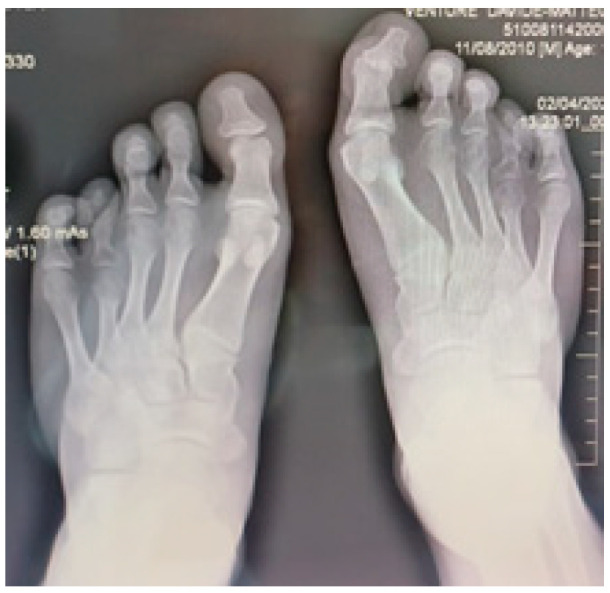
Radiological image of both feet shows bilateral hypoplasia of the fourth and fifth metatarsal bones and phalanges.

Radiographic examination confirmed bilateral hypoplasia of the metacarpals and phalanges of digits IV and V of the hands (Figure 4), and hypoplasia of the metatarsals and phalanges of digits IV and V of the feet (Figure 5).

#### 2.1.1. Family Investigation and Segregation Analysis

Both parents underwent clinical evaluation and showed no morphological abnormalities; neuropsychological assessment revealed normal intellectual function and behaviour. Genetic testing of the patient’s mother for the RBPJ and CACNA1A variants was negative. Genetic testing of the father was not performed because he declined to participate.

The patient’s one-year-old half-brother (same mother, different father) was evaluated due to recurrent allergic skin manifestations and microtia. Whole-exome sequencing did not identify pathogenic variants in RBPJ or CACNA1A in this individual.

Segregation analysis was therefore incomplete. While maternal inheritance was excluded, de novo occurrence of the variants could not be formally confirmed due to the unavailability of paternal genetic testing.

#### 2.1.2. Neurological Findings

The patient did not exhibit the classical neurological phenotypes typically associated with CACNA1A-related disorders, such as cerebellar ataxia, vertigo, nystagmus, hemiplegic migraine, or epileptic encephalopathy. There was no reported history of episodic gait instability, migraine, or vertiginous episodes.

The patient experienced a single afebrile seizure at the age of one year. Subsequent neurological investigations, including electroencephalography (EEG) and brain magnetic resonance imaging (MRI), were normal, with no structural abnormalities identified, particularly in the cerebellum. Neurological examination revealed no ocular motor abnormalities, including nystagmus, and no motor coordination deficits. At the time of the most recent evaluation, the patient had no recurrent seizures and was not receiving antiepileptic treatment. The neurological phenotype was instead characterised by mild intellectual disability and neurobehavioral features within the autism spectrum, which represent the predominant clinical manifestations.

#### 2.1.3. Laboratory Tests

Complementary laboratory investigations included metabolic and genetic assessments. Serum homocysteine, measured enzymatically, was elevated at 19 µmol/L (normal ≤ 12 µmol/L). Vitamin B12 concentration was 300 pg/mL (normal 270–1132 pg/mL), and methionine was within normal limits at 2.3 mg/L (normal 1–7 mg/L). Methylmalonic acid (MMA), determined by gas chromatography–mass spectrometry, was elevated at 46 µg/L (normal 9–32 µg/L), indicating functional vitamin B12 deficiency (Table 1).

The combination of elevated MMA levels with borderline serum vitamin B12 concentrations was consistent with a possible functional vitamin B12 deficiency. At the time of this report, vitamin supplementation (vitamin B12, folate, or vitamin B6) had not yet been initiated, and therefore follow-up biochemical testing (repeat methylmalonic acid or homocysteine levels) and assessment of clinical response were not available.

### 2.2. Clinical Timeline

To improve clarity and ensure chronological consistency of the clinical history, a structured timeline of key events from birth to follow-up evaluation is provided below.

The patient was born at term following an uncomplicated pregnancy and delivery, with shortening of the fourth and fifth digits of both hands and feet noted at birth. During infancy, at approximately 12 months of age, he experienced an episode of afebrile seizures. Throughout early childhood, neurodevelopmental concerns became evident, including behavioural dysregulation, attention deficit, learning difficulties, and mild intellectual functioning. The patient was first referred for genetic evaluation at the age of 10 years due to persistent skeletal abnormalities and neurodevelopmental concerns. Whole exome sequencing was performed thereafter as part of the diagnostic workup. A comprehensive clinical, radiological, and genetic re-evaluation was conducted at the age of 14 years, which forms the basis of the current report.

### 2.3. Genetic Investigation

Given the atypical combination of terminal limb anomalies and neurobehavioral features, whole-exome sequencing (WES) was performed at the age of 10 years to provide a comprehensive genetic assessment and identify potential pathogenic variants underlying the observed phenotype. PCR/Real-Time PCR (LightCycler) testing identified two MTHFR polymorphisms—C677T (rs1801133) and A1298C (rs1801131)—both in heterozygous form. Whole-exome sequencing was subsequently performed in the proband, identifying a heterozygous missense variant in RBPJ (NM_005349.3:c.505A>G; p.(Lys169Glu); rs387907271). WES was conducted using an Illumina platform (Illumina, Inc., San Diego, CA, USA). Genomic DNA was enzymatically fragmented and tagged with Illumina-compatible adapter sequences, and libraries were paired-end sequenced, achieving a mean coverage depth of approximately 30×, with ≥20× coverage for more than 98% of the targeted bases. Sequencing reads were aligned to the Genome Reference Consortium Human Build 37 (GRCh37/hg19) and to the revised Cambridge Reference Sequence (rCRS; NC_012920) of human mitochondrial DNA. Variant calling for single-nucleotide variants (SNVs), insertions/deletions (indels), and copy number variants (CNVs) was performed using the Illumina DRAGEN pipeline (version 4.4.6), Manta (v1.6.0), and CENTOGENE’s validated in-house algorithms.

Variant analysis focused on coding exons and flanking ±10 intronic nucleotides of genes with established gene–phenotype associations based on OMIM^®^ information. Variants with a minor allele frequency < 1% in gnomAD, as well as disease-associated variants reported in HGMD^®^, ClinVar, or the CENTOGENE in-house biodatabase, were evaluated. All potential modes of inheritance were considered, and available family history and clinical information were incorporated into variant interpretation.

Sequence variants were classified into five categories (pathogenic, likely pathogenic, variant of uncertain significance [VUS], likely benign, and benign) according to ACMG/AMP guidelines, with additional consideration of ClinGen recommendations. CNVs were classified according to ACMG/ClinGen guidelines.

Clinically relevant variants with low sequencing quality and/or ambiguous zygosity were confirmed by orthogonal methods, ensuring a reported specificity of >99.9%. Mitochondrial variants were reported for heteroplasmy levels of ≥15%, and CNV detection demonstrated a sensitivity exceeding 95%. Screening for uniparental disomy (UPD) was performed using a dedicated algorithm targeting clinically relevant chromosomal regions (6q24, 7, 11p15.5, 14q32, 15q11–q13, 20q13, and 20).

WES identified two relevant heterozygous variants: RBPJ (NM_005349.3:c.505A>G; p.(Lys169Glu)) associated with autosomal dominant Adams–Oliver syndrome type 3, and CACNA1A (NM_023035.2:c.5032C>T; p.(Arg1678Cys)) previously reported in association with episodic ataxia type 2, familial hemiplegic migraine, developmental and epileptic encephalopathy (DEE42), paroxysmal dystonia, and neuropsychiatric disorders.

#### Genetic Findings/Variant Interpretation

The CACNA1A variant (NM_000068.4:c.5032C>T; p.Arg1678Cys) was classified as likely pathogenic according to ACMG/AMP criteria. This classification was supported by its absence from population databases (PM2), multiple in silico predictions indicating a deleterious effect (PP3), previous reports in affected individuals with epilepsy and hemiplegic migraine-related phenotypes (PS4_supporting) [11], and partial phenotypic overlap with the known CACNA1A-associated neurodevelopmental spectrum (PP4). The variant is reported in ClinVar as Likely pathogenic (Variation ID: 196769). Segregation analysis was incomplete: the variant was absent in the mother, while paternal testing was not available; therefore, de novo occurrence could not be confirmed.

The RBPJ variant (NM_005349.4:c.505A>G; p.Lys169Glu) was classified as pathogenic based on ACMG/AMP criteria, including previous reports in individuals with Adams–Oliver syndrome type 3 (PS4) [12], absence from population databases (PM2), and a highly specific phenotype consistent with RBPJ-related AOS3 (PP4). This variant is listed in ClinVar as Pathogenic (Variation ID: 37054). Maternal testing was negative, and paternal testing was not available, resulting in incomplete segregation data.

A systematic evaluation of the major and minor diagnostic criteria for Adams–Oliver syndrome was performed, and the findings are summarised in Table 2.

Table 3 summarises the key genotype–phenotype correlations observed in this patient. The pathogenic RBPJ variant is associated with the limb malformations characteristic of Adams–Oliver syndrome type 3, while the heterozygous CACNA1A variant may act as a modifying factor contributing to the neurodevelopmental phenotype, including mild intellectual disability and a history of an early, isolated seizure with normal EEG findings. The common MTHFR polymorphisms (C677T and A1298C), which are highly prevalent in the general population, are reported in relation to the observed metabolic findings (elevated homocysteine and methylmalonic acid) and are interpreted as potential modifiers rather than primary pathogenic drivers.

## 3. Discussion

In this case, we identified a heterozygous pathogenic variant in RBPJ—the key nuclear mediator of Notch signalling—in a boy presenting with terminal transverse limb defects consistent with Adams–Oliver syndrome type 3 (AOS3). The same variant, c.505A>G (p.Lys169Glu), has been previously reported in one of the two families described initially by Hassed et al. [13], where affected individuals displayed variable expression ranging from mild digital anomalies to more extensive limb reductions and intellectual disability. The present patient shows a similarly heterogeneous pattern, with bilateral hypoplasia of the fourth and fifth digits of both hands and feet, but without scalp involvement, a feature absent in only a minority of clinically confirmed AOS cases [14,15]. This finding provides further support for the notion that ACC is not obligatory for diagnosis when a pathogenic RBPJ variant is present. Microcephaly, scalp defects, and skull ossification defects are often present in the clinical picture, as are central nervous system malformations. In some cases, incomplete ossification of the skull underlying the affected scalp area may occur, resulting in scarring and permanent alopecia [16].

The classification of AOS has evolved from an initial three-type phenotypic system [17] to a genetic framework consisting of six subtypes (AOS1–AOS6), involving both autosomal dominant (ARHGAP31, RBPJ, NOTCH1, DLL4) and autosomal recessive (DOCK6, EOGT) genes [15,18,19]. Among dominant forms, AOS3 related to RBPJ tends to show milder limb anomalies than DOCK6-associated AOS2, and usually lacks congenital heart disease—a pattern consistent with our patient’s presentation. RBPJ is the central transcriptional mediator of the Notch pathway [20,21,22,23]. The present phenotype matches the known physiologic effects of RBPJ malfunction. Along with AOS3, the patient carries a heterozygous CACNA1A variant (c.5032C>T; p.Arg1678Cys), a variant associated with marked phenotypic variability. CACNA1A-related disorders include episodic ataxia type 2 (EA2), familial hemiplegic migraine type 1 (FHM1), developmental and epileptic encephalopathy (DEE42), and various neuropsychiatric manifestations [7]. Loss-of-function (LOF) variants are typically associated with mild to moderate intellectual disability, whereas gain-of-function (GOF) variants tend to produce more severe neurodevelopmental impairment [24]. The variant identified here lies in a region previously associated with LOF effects. Reports of CACNA1A haploinsufficiency describe individuals with intellectual disability, autism spectrum traits, epilepsy, and subtle cerebellar signs [25]. Although the patient’s neurocognitive profile could partly reflect the consequences of chronic psychosocial and developmental factors, the presence of this variant may contribute to his mild cognitive performance, attention difficulties, and behavioural dysregulation.

The patient also harbours the common MTHFR polymorphisms C677T and A1298C, both in heterozygous form. These variants are highly prevalent in the general population and are generally regarded as polymorphisms with modest or uncertain clinical impact, rather than pathogenic variants. Although reduced MTHFR activity has been associated with elevated homocysteine levels, and elevated methylmalonic acid levels, despite serum vitamin B12 concentrations within the normal range, heterozygosity for these variants alone rarely results in clinically significant metabolic or neuropsychiatric disease [26,27]. Associations between MTHFR polymorphisms and neuropsychiatric conditions such as autism spectrum disorder, ADHD, or mood disorders have been reported in some studies, but effect sizes are typically small and findings remain inconsistent [28,29,30,31,32,33,34,35]. Accordingly, the presence of these variants in the patient should be interpreted with caution and not as a causal explanation for the neuropsychiatric phenotype.

The patient’s MTHFR genotype is best viewed as a modifying factor that may increase vulnerability in combination with other genetic or environmental influences [36,37,38]. The patient’s biochemical findings, including elevated homocysteine and methylmalonic acid levels, likely reflect a multifactorial metabolic imbalance rather than a direct consequence of MTHFR heterozygosity alone. While correction of hyperhomocysteinemia through vitamin supplementation has been associated with neuropsychiatric improvement in selected contexts [39,40,41], responses are variable and do not establish a direct causal relationship.

The present case further illustrates the broad phenotypic spectrum associated with Adams–Oliver syndrome and related neurodevelopmental abnormalities. As observed in previously documented AOS cohorts, clinical severity may vary substantially even among individuals carrying the same pathogenic variant [42]. This variability is not limited to AOS; similar patterns have been described in other multisystem congenital disorders. The importance of re-examining patients at different developmental stages has also been highlighted in other disorders. A study evaluating OFDI syndrome from childhood to adolescence demonstrated that phenotypic expression can change significantly from early childhood to adolescence, reinforcing the clinical value of longitudinal assessment [43]. Such findings underscore the relevance of follow-up beyond infancy, where delayed or progressive manifestations may emerge over time.

The differential diagnosis of Adams–Oliver syndrome (AOS) requires careful clinical and genetic evaluation, as several congenital disorders can present with overlapping limb, scalp, or vascular abnormalities [44]. Aplasia cutis congenita (ACC) in isolation must be distinguished from AOS, particularly when scalp defects occur without associated limb anomalies [45,46]. Amniotic band sequence is another important consideration, as it may produce transverse limb defects that mimic those seen in AOS, though typically in an asymmetric, non-hereditary pattern [47]. Conditions affecting distal limb development, such as Adams–Oliver-like phenotypes related to DOCK6, EOGT, or NOTCH1 variants, and other acrofacial dysostoses, may also resemble AOS but frequently include facial or craniofacial malformations not observed in classic forms of the syndrome. Vascular anomalies and cutis marmorata telangiectatica congenita (CMTC) may occur as isolated entities or in combined vascular disorders, and should be excluded when present without congenital scalp or limb defects [48]. In patients with neurological involvement or intellectual disability, syndromes such as CACNA1A-related neurodevelopmental disorders or other genetic limb-brain conditions should be considered. Ultimately, confirmation of AOS—particularly AOS3—relies on correlating characteristic phenotypic features with pathogenic variants in genes of the Notch signalling pathway, most commonly RBPJ [49].

In the present case, the heterozygous RBPJ variant is considered the primary candidate variant with the highest diagnostic relevance, based on its established role in Notch signalling, its known association with Adams–Oliver syndrome, and prior reports linking RBPJ pathogenic variants to autosomal dominant forms of the disorder. The RBPJ variant is the one that best explains the patient’s core clinical features.

By contrast, the CACNA1A and MTHFR variants are interpreted as secondary findings with potential modifying or contributory effects rather than as independent monogenic causes. The CACNA1A missense variant, while classified as likely pathogenic based on in silico predictions and rarity, lacks functional validation and is not accompanied by a phenotype classically associated with CACNA1A-related disorders in this patient. Consequently, its contribution, if any, is considered modulatory rather than causative. Similarly, the MTHFR C677T and A1298C variants represent common polymorphisms with modest biochemical effects and are discussed exclusively in the context of metabolic modulation, without implying a primary pathogenic role. Overall, the clinical phenotype in this case is best explained by a model in which a single primary pathogenic variant (RBPJ) accounts for the core disease manifestation. In contrast, additional genetic variants may contribute to phenotypic variability or severity. This framework aligns with current clinical genomics practice and helps prevent overinterpretation of secondary findings.

Several limitations should be acknowledged. As a single case report, the findings cannot be generalised to the broader AOS or neurodevelopmental populations. No functional assays were performed to assess the impact of the RBPJ or CACNA1A variants on protein function; therefore, variant interpretation relies on genetic, clinical, and literature-based evidence. In addition, segregation analysis was incomplete, as not all family members were available for genetic testing, precluding confirmation of de novo status. Environmental, psychosocial, and epigenetic factors may have contributed to the neurodevelopmental profile but could not be systematically evaluated. Future studies are needed to clarify genotype–phenotype correlations in AOS3 and to understand better how additional variants, such as CACNA1A or common MTHFR polymorphisms, may act as modifiers of neurological outcomes.

## 4. Conclusions

In summary, this report describes, for the first time, the presence of an RBPJ gene variant consistent with AOS3 in a patient who also carries a pathogenic variant in CACNA1A and common functional polymorphisms in MTHFR. Molecular analysis is essential for establishing an accurate genetic diagnosis, clarifying genotype–phenotype correlations, assessing recurrence risk, and guiding appropriate genetic counselling.

## Figures and Tables

**Table 1:** Metabolic and biochemical testing.

Biochemical Marker	Result	Normal Range	Interpretation
Serum homocysteine	19 µmol/L	≤12 µmol/L	Elevated
Vitamin B12	300 pg/mL	270–1132 pg/mL	Borderline low
Methionine	2.3 mg/L	1–7 mg/L	Normal
Methylmalonic acid	46 µg/L	9–32 µg/L	Elevated

**Table 2:** Major and minor diagnostic criteria for Adams–Oliver Syndrome (AOS) and evaluation of the case.

Category	Diagnostic Criterion	Present Case
Major criteria	Aplasia cutis congenita (ACC)	Absent
	Terminal transverse limb defects (TTLD)	Present
	Positive family history	Absent
Minor criteria	Cutis marmorata telangiectatica congenita (CMTC)	Absent
	Vascular anomalies	Absent
	Congenital heart defects	Absent
	Neurological abnormalities	Absent *
	Craniofacial anomalies (depressed nasal bridge and low-set ears)	Present
	Ocular anomalies (myopia and astigmatism)	Present
Genetic findings	Pathogenic/likely pathogenic variant in AOS-related genes	Present
WES analysis	Whole-exome sequencing	Positive

* Neurological abnormalities are listed as a minor AOS criterion; although the patient had a history of seizures and neurodevelopmental issues, these findings are considered more consistent with the coexisting CACNA1A pathogenic variant rather than a primary AOS manifestation.

**Table 3:** Genotype–phenotype summary.

Category	Clinical Findings	Gene/Variant
Genotype	Heterozygous pathogenic variant	RBPJ (c.505A>G; p.Lys169Glu)
	Heterozygous variant	CACNA1A (c.5032C>T; p.Arg1678Cys)
	Common polymorphisms	MTHFR C677T and A1298C (heterozygous)
Limb anomalies	Bilateral hypoplasia of the 4th and 5th digits of hands and feet	RBPJ
Neurodevelopmental	Mild intellectual disability (IQ 69)	CACNA1A (possible modifier)
Seizures/EEG	Single afebrile seizure at 1 year of age; EEG normal; no recurrent seizures	CACNA1A
Metabolic	Elevated homocysteine and methylmalonic acid; borderline vitamin B12	MTHFR (possible modifier)

## Data Availability

The data are not publicly available due to privacy and ethical restrictions, as they contain information that could compromise patient confidentiality.

## Abbreviations

The following abbreviations are used in this manuscript:

ACC	Aplasia Cutis Congenita
AD	Autosomal Dominant
ADHD	Attention-Deficit/Hyperactivity Disorder
AOS	Adams–Oliver Syndrome
AOS1–AOS6	Adams–Oliver Subtypes 1–6
AR	Autosomal Recessive
ASD	Autism Spectrum Disorder
CACNA1A	Calcium Voltage-Gated Channel Subunit Alpha 1A
CMTC	Cutis Marmorata Telangiectatica Congenita
CNV	Copy Number Variation
DEE42	Developmental and Epileptic Encephalopathy Type 42
DLL4	Delta-like Ligand 4
DOCK6	Dedicator of Cytokinesis 6
EOGT	EGF Domain-Specific O-Linked N-Acetylglucosamine Transferase
FHM1 GOF	Familial Hemiplegic Migraine Type 1 Gain-of-function
GDD	Global Developmental Delay
Hcy LOF	Homocysteine Loss-of-function
MIM	Mendelian Inheritance in Man
MMA	Methylmalonic Acid
MTHFR	Methylenetetrahydrofolate Reductase
NICD	Notch Intracellular Domain
NOTCH1	Notch Receptor 1
OFC	Occipito-Frontal Circumference
PCR	Polymerase Chain Reaction
RBPJ	Recombination Signal Binding Protein for Immunoglobulin κ J Region
SAM	S-Adenosylmethionine
SD	Standard Deviation
SHARP	SMRT/HDAC-Associated Repressor Protein
SNV	Single Nucleotide Variant
THF	Tetrahydrofolate
TTLD	Transverse Terminal Limb Defects
WES	Whole Exome Sequencing
WGS	Whole Genome Sequencing
